# *Pseudomonas aeruginosa* Septic Arthritis and Osteomyelitis after Closed Reduction and Percutaneous Pinning of a Supracondylar Humerus Fracture: A Case Report and Review of the Literature

**DOI:** 10.1155/2017/8721835

**Published:** 2017-11-14

**Authors:** Adam M. Wegner, John C. Wuellner, Brian M. Haus

**Affiliations:** University of California Davis Medical Center, Sacramento, CA, USA

## Abstract

Infectious complications of closed reduction and percutaneous pinning of supracondylar humerus fractures are exceedingly rare. Although postoperative *Pseudomonas* infection is a feared complication associated with noncompliance and a wet cast, there are no reports in the literature of this occurring. We present the devastating complication of a pediatric patient who developed *Pseudomonas aeruginosa* subperiosteal abscess, osteomyelitis, and elbow septic arthritis after presenting to the clinic multiple times with a wet cast after closed reduction and percutaneous pinning of a supracondylar humerus fracture. We describe the treatment course for this patient, followed by the sequelae of posterolateral rotary instability. This case not only confirms that patients can get *Pseudomonas* infections if they get their cast wet but also stresses the importance of patient communication and compliance in preventing unfortunate complications.

## 1. Introduction

Supracondylar humerus fractures (SCHFXs) are the most common elbow fracture in children, accounting for a third of all extremity fractures [1, 2]. They occur most frequently between the ages of 4 and 8 years and are typically sustained through falls onto an outstretched hand or extended arm, resulting in an extension-type fracture [3, 4]. Closed reduction and percutaneous pinning (CRPP) has become the mainstay of surgical treatment for Gartland type II and III injuries [5].

Complications associated with CRPP of SCHFXs are rare, occurring in 2.4–7.4% of cases. The most common complications include migration of pins with subcutaneous retraction, pin tract infection, and loss of fracture reduction [6]. Deep infection after CRPP is exceedingly rare. In a retrospective cohort of patients undergoing CRPP for SCHFXs reported by Bashyal et al., there was no osteomyelitis or septic arthritis in 287 (0%) surgically treated Type II and only 1 of 288 (0.3%) in Type III [6].

A recent retrospective analysis covering 17 years of all pediatric Kirschner wire infections requiring hospitalization at an institution reported 12 patients of 884 (1.3%) sustaining this complication [7]. Operatively treated SCHFXs were the most commonly infected. Four of the five reported cases were associated with a wet cast. All of the cases cultured were associated with Gram-positive cocci, specifically *Staphylococcus* and *Streptococcus* organisms.

In this case report, we describe the first reported case of *Pseudomonas aeruginosa* subperiosteal abscess, osteomyelitis, and septic arthritis following CRPP of a Type III SCHFX.

## 2. Case Presentation

**Figure 1:**
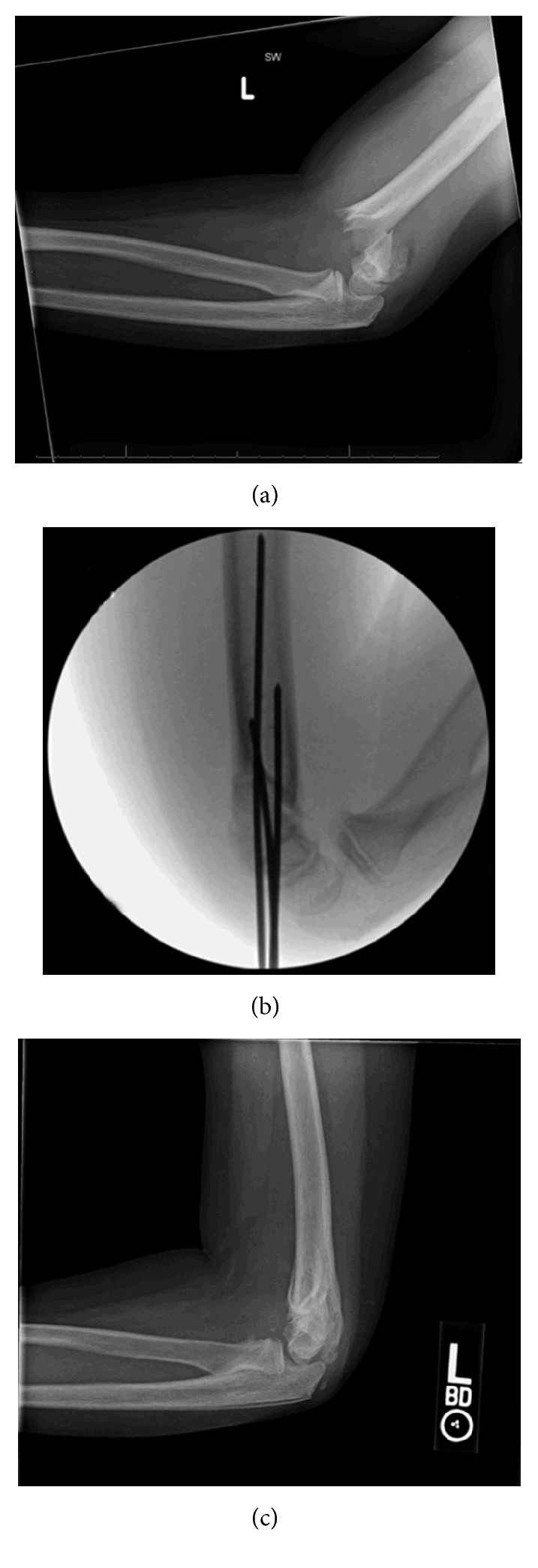
Plain films of the left elbow immediately after injury in the emergency department (a), intraoperative fluoroscopic film showing reduction and pin placement (b), and follow-up plain film imaging at 12 weeks after surgery (c).

A 7-year-old girl was transferred to the orthopaedic service from an outside hospital with a left elbow SCHFX after jumping off a one-storey building onto a trampoline, landing on her outstretched arms. On physical exam, there was moderate diffuse edema about the elbow with no pucker sign. She was neurovascularly intact, and radiographic evaluation demonstrated a left Type III SCHFX (Figure 1(a)).

After the arm was prepped with chloraprep and weight-based prophylactic cefazolin was administered, a CRPP was performed. Three divergent lateral pins were cut outside of the skin by one centimeter and then bent to 90°. A sterile felt was placed to protect the exposed pins from edema, and the pins were then covered in sterile cotton cast padding (Figure 1(b)). Finally, a bivalved long arm cast was placed, and the patient and mother were instructed to keep the cast clean and dry. The patient returned to the clinic on postoperative day three for follow-up radiographic imaging, which showed maintained alignment. The cast was overwrapped, and again the patient and her mother were instructed to keep cast clean and dry. At that point, they were instructed to follow-up in four weeks for repeat radiographs, where cast and pin removal would be performed if adequate healing had occurred.

One week later (10 days post-op), the patient returned with her mother with a dirty, wet, and loose cast. The pin sites appeared clean at this time. After replacing the long arm cast, the mother and patient were again instructed on the importance of keeping the cast clean and dry. Again, one week later, the patient and her mother returned with a wet cast. The mother reported that due to the recent hot weather and broken home air conditioning, the patient had gone swimming in a friend's pool after wrapping the cast in a plastic bag. Upon removal of the cast, there was granulation tissue noted at the pin sites but no erythema, purulent drainage, or skin maceration. The cast smelled of mildew. The patient did not have any constitutional symptoms and was afebrile. The mother was extensively educated on the importance of keeping the cast dry.

The patient returned two weeks later for the four-week postoperative visit for radiographic evaluation and pin removal. The cast appeared to have gotten wet again. Upon removal, there was a small amount of serous fluid draining from the pin sites. The patient had a buildup of significant granulation tissue, no erythema, fluctuance, skin maceration, or increased pain with elbow motion and was afebrile. Pins were pulled at that point, as the fracture appeared to be radiographically healed, and granulation tissue was cauterized with several silver nitrate sticks.

The patient returned to the clinic two weeks later with a large elbow effusion, bloody purulent fluid draining from a sinus tract, and radiographs with the radiocapitellar joint dislocated posterolaterally and periosteal and cortical reactions (Figure 1(c)). The patient was afebrile, with a CRP of 3.9 (0–0.8), ESR of 71 (0–13), and WBC of 13.4 (5.0–14.5). Empiric vancomycin and cefazolin were initiated for presumed Gram-positive infection, magnetic resonance imaging (MRI) was obtained, and she was admitted to the hospital for surgical I&D.

**Figure 2:**
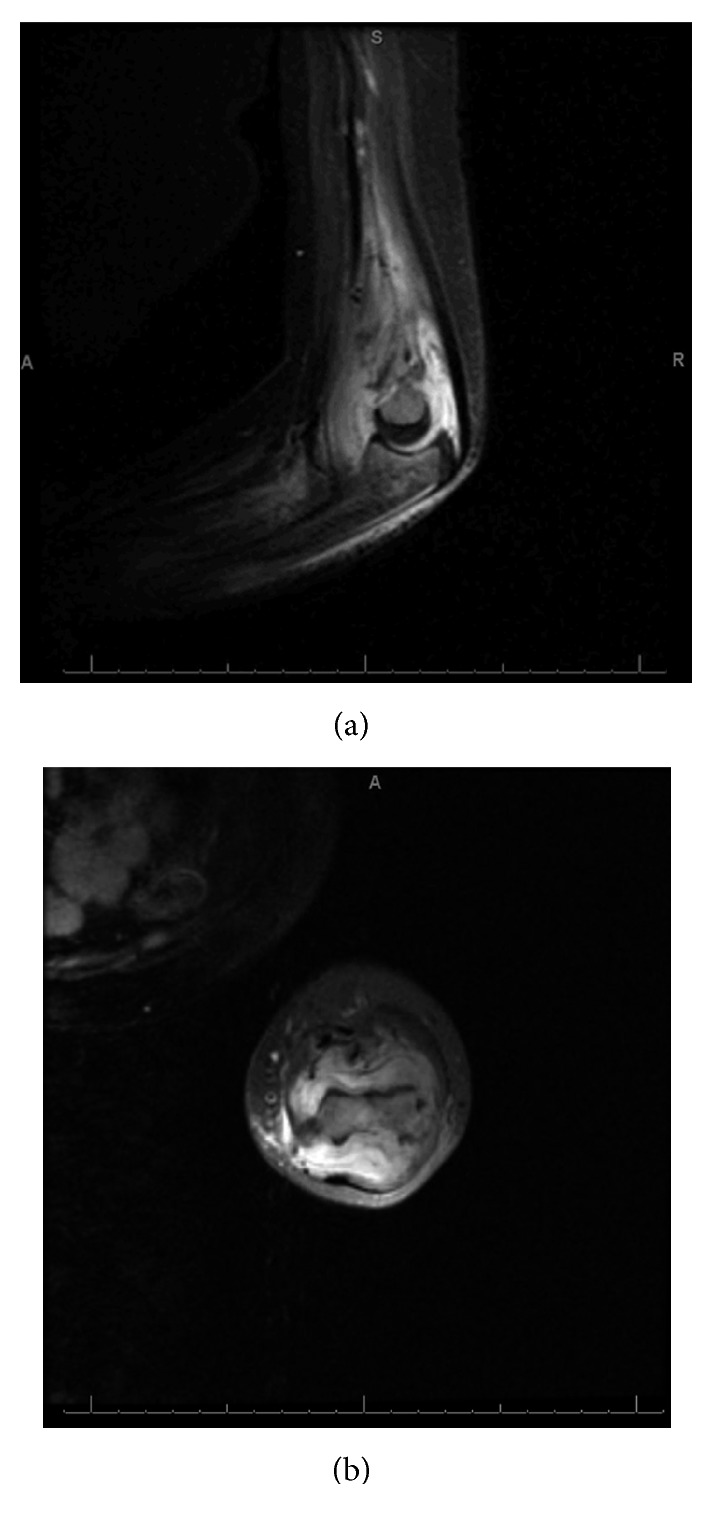
IR sagittal MRI of the left elbow showing a large loculated joint effusion with tracking up the posterior aspect of the humerus (a). T2 fat-suppressed axial MRI of the left distal humerus showing joint effusion (b).

The MRI showed a subperiosteal abscess, osteomyelitis, and a large elbow joint effusion (Figure 2). She was subsequently taken to the operating room for incision and drainage of her left elbow and distal humeral subperiosteal abscess. The capsule was distended and filled with loculated pus which also tracked up the posterior aspect of the humerus and 1.5 cm down the radius, where it also disrupted the annular ligament and lateral collateral ligament. Several samples were sent for culture. On exam under anesthesia, she had gross posterolateral rotatory instability (PLRI). She was only stable in flexion to 90° with full pronation, so was splinted in that position.

Two days later, after her CRP trended up to 7.1, her elbow was washed out again and splinted in the same position. Cultures grew *Pseudomonas aeruginosa* that morning, and the patient was switched to intravenous piperacillin/tazobactam. Two days after the second I&D and antibiotic switch, her CRP decreased to 0.9, the WBC normalized, and she remained afebrile. She was then started on intravenous levofloxacin after the minimum inhibitory concentrations (MICs) returned. Pediatric Infectious Disease recommended a 6-week course, with transition to oral as an outpatient. Five days after the initial debridement, the patient's CRP had decreased to 0.9 from a high of 7.1 (normal range 0–0.8). On the 6th day, the patient returned to the OR for final I&D, annular ligament repair, LCL repair, and closure. The wound appeared to be clean with normal granulation tissue and no purulent material, although no culture or stat gram stain was obtained at that time. The elbow was stable in all positions except for a few millimeters of subluxation with supination in extension. The arm was casted in 90° of flexion and full pronation, with a plan to switch her to a hinged elbow brace in 3 weeks to start motion to prevent stiffness. At 1 week after discharge, the wound was healing well without signs of infection, and the patient was again placed in long arm cast. She followed up again two weeks later and her cast was removed. She had no pain, and her incision was healing well with no signs of infection. She had 45° of passive extension, 110° of passive flexion, 0° of supination, and 45° of pronation. The fracture was radiographically healed. She was placed in a hinged elbow brace and instructed to perform passive assisted extension and flexion. She followed up for her final visit in three weeks with similar elbow range of motion. Her sutures were removed, and she was referred to a pediatric complex elbow reconstruction surgeon for further care. She was also referred to occupational therapy to help assist with increasing her range of motion.

## 3. Discussion

CRPP and casting of SCHFXs typically result in excellent outcomes with minimal complications. Rates of infection after CRPP of SCHFX are very low, with several studies reporting overall infection rates of approximately 2.3% [8, 9]. Deep infection, defined as septic arthritis or osteomyelitis, is even more rare, with rates of 0.3%–0.5% [6–9]. However, noncompliance with instructions can place patients at increased risk for rare complications, as was observed in this case. Patient and family education on the postoperative course is arguably the most important aspect of surgery.

The most commonly isolated pathogen in hardware-associated infections in orthopaedics is methicillin-resistant *Staphylococcus aureus* (MRSA), followed by *Streptococcus pyogenes*, *Staphylococcus epidermidis*, and *Pseudomonas aeruginosa* [7, 10]. This suggests that if a surgical site infection is suspected, such as in our patient, then antibiotic coverage should be provided for *Staphylococcus* and considered for *Pseudomonas*. In this patient, vancomycin and cefazolin were chosen, covering MRSA and most Gram-positive cocci with few Gram-negative bacilli, but not including *Pseudomonas*. It likely would not have impacted her condition to have chosen an agent that covered *Pseudomonas*, given how advanced her infection was at presentation; however, providing coverage for *Pseudomonas* would be very important in a patient with a more limited infection.

Pre- and postoperative antibiotics and pin site care have been used in attempts to further reduce the rate of infection in CRPP. Despite the common use of preoperative antibiotics, there are limited data to support its practice. A retrospective analysis of 2330 minimally invasive procedures in pediatric orthopaedic surgery was unable to find any reduction in the rate of complicated surgical site infection requiring reoperation in a group that received preprocedure antibiotics versus those that did not, although this was not statistically significant due to their exceedingly low SSI rate of 0.0008% in the no antibiotic group [11]. Our patient received preoperative cefazolin which likely did not negatively affect her outcome but also provided little to no benefit.

There have been no studies showing a benefit of postoperative antibiotics in CRPP of SCHFXs, despite its fairly common implementation. Our patient did not receive postoperative antibiotics following her procedure. Schroeder et al. retrospectively found a surgical site infection rate of 1.8% in 618 CRPP of SCHFX regardless of whether postoperative antibiotics were administered [9]. Iobst et al. also reported that the majority of their population, 67%, did not receive any antibiotics, pre or post, and they reported no infections in their treatment population of 304 patients [8]. Pin site care after CRPP of SCHFX negatively impacts pin site infection rates. Kao et al. reported an increase in pin site infection rate in the daily pin site care group. They also noted that the process was painful for the patient and there was a notable increase in the number of telephone consultations [12].

Although a majority of cases of deep pin site infections after SCHFX are associated with poor compliance or deviations from the surgeon's recommendations, such as a wet splint/cast wet or a missed follow-up appointment, a *Pseudomonas* infection has never before been reported. Patients and their caretakers must be educated on the complications that may arise if they do not follow the treatment plan. Ultimately, as healthcare providers, we are responsible for adequately and effectively communicating care instructions to our patients and their caretakers, but we are not able to make them comply with those directions. Some amount of responsibility must be taken by them in the implementation phase.

Long-term sequelae of deep musculoskeletal infections in the pediatric population are very significant. Angular limb deformity and limb length discrepancy are complications of osteomyelitis, and osteonecrosis and joint stiffness are the main complications of septic arthritis [8]. Due to our patient's extensive infection, she is at increased risk to experience more than one of these sequelae. At the end of her treatment course, despite work to increase her range of motion, she was still very stiff, most notably lacking 45° of extension. Therefore, she was referred to a pediatric elbow reconstruction expert for further management.
